# Usability Evaluations of Mobile Mental Health Technologies: Systematic Review

**DOI:** 10.2196/15337

**Published:** 2020-01-06

**Authors:** Yavuz Inal, Jo Dugstad Wake, Frode Guribye, Tine Nordgreen

**Affiliations:** 1 Department of Information Science and Media Studies University of Bergen Bergen Norway; 2 NORCE Norwegian Research Centre Bergen Norway; 3 Psychiatric Division Haukeland University Hospital Bergen Norway

**Keywords:** systematic review, mobile, mHealth, mental health, usability evaluation

## Abstract

**Background:**

Many mobile health (mHealth) apps for mental health have been made available in recent years. Although there is reason to be optimistic about their effect on improving health and increasing access to care, there is a call for more knowledge concerning how mHealth apps are used in practice.

**Objective:**

This study aimed to review the literature on how usability is being addressed and measured in mHealth interventions for mental health problems.

**Methods:**

We conducted a systematic literature review through a search for peer-reviewed studies published between 2001 and 2018 in the following electronic databases: EMBASE, CINAHL, PsycINFO, PubMed, and Web of Science. Two reviewers independently assessed all abstracts against the inclusion and exclusion criteria, following the Preferred Reporting Items for Systematic Review and Meta-Analysis guidelines.

**Results:**

A total of 299 studies were initially identified based on the inclusion keywords. Following a review of the title, abstract, and full text, 42 studies were found that fulfilled the criteria, most of which evaluated usability with patients (n=29) and health care providers (n=11) as opposed to healthy users (n=8) and were directed at a wide variety of mental health problems (n=24). Half of the studies set out to evaluate usability (n=21), and the remainder focused on feasibility (n=10) or acceptability (n=10). Regarding the maturity of the evaluated systems, most were either prototypes or previously tested versions of the technology, and the studies included few accounts of sketching and participatory design processes. The most common reason referred to for developing mobile mental health apps was the availability of mobile devices to users, their popularity, and how people in general became accustomed to using them for various purposes.

**Conclusions:**

This study provides a detailed account of how evidence of usability of mHealth apps is gathered in the form of usability evaluations from the perspective of computer science and human-computer interaction, including how users feature in the evaluation, how the study objectives and outcomes are stated, which research methods and techniques are used, and what the notion of mobility features is for mHealth apps. Most studies described their methods as trials, gathered data from a small sample size, and carried out a summative evaluation using a single questionnaire, which indicates that usability evaluation was not the main focus. As many studies described using an adapted version of a standard usability questionnaire, there may be a need for developing a standardized mHealth usability questionnaire.

## Introduction

### Background

Digital technology for screening, treatment, and management of mental health issues has proliferated in recent years, and a substantial share of these applications is implemented on mobile devices [1]. Firth and Torous [2] argue that mobile technologies are particularly suitable to provide services for behavioral health, such as psychiatry, because of the opportunities for capturing patient behavior, for example, through ecological momentary assessment [3] and providing real-time support, given the omnipresence of mobile devices. According to a 2015 report on mobile health (mHealth) adoption, there were 165,000 mHealth apps available on Google Play and iTunes Store, a third of which focused on dieting, wellness, and exercise, and about a quarter of these concerned disease treatment. One-third of the disease-specific apps were for mental health [4].

The World Health Organization (WHO) [5] acknowledges the potential in mHealth apps for meeting the challenges in reaching universal health coverage, provided the apps are evidenced. This involves critically scrutinizing their “benefits, harms, acceptability, feasibility, resource use, and equity considerations.” There are different approaches to scientifically assessing the effect, utility, and usefulness of mHealth apps. From the perspective of medicine and psychiatry, the acceptable way of measuring the effects on mental health is through randomized controlled trials [3,6]. From the perspectives of computer science and human-computer interaction (HCI), a well-established approach to the assessment of technology is to evaluate their usability. As a science, usability is grounded not only in the social and behavioral sciences but also in the science of design [7]; however, poor usability and lack of user-centered design have been described as 2 of the reasons for low engagement with mHealth apps [8], and attrition is considered a generic problem in mHealth [9].

Usability is defined by Nielsen [10] as a “quality attribute that assesses how easy interfaces are to use.” Usability evaluation has the purpose of gaining understanding of how easy it is to use an interface, and it is an essential part of systems development [11]. There can be different motivations behind usability evaluations, such as establishing evidence that the interface is usable (summative) or informing the redesign and improvement of the interface (formative). Systems with poor usability can lead to situations of low goal-achievement efficiency or the technology not being used or being rejected. Usability evaluation methods are divided into inspection or heuristic methods and methods that are based on input from user representatives. Usability evaluations are usually undertaken in relation to an interaction design process. In HCI, there is an ideal that the results from the evaluation are used to inform the redesign of the evaluated interface but according to Nørgaard and Hornbæk [12], this is often not the case as, rather surprisingly, the evaluation and redesign often occur independently of each other.

Recently, the scope of usability evaluation has shifted from usability engineering to the more encompassing task of evaluating user experience, including user emotions, values, and motivations [13]. At the same time, digital technology is increasingly being directed at the private and public spheres of the users [14], spreading from the “workplace to our homes, everyday lives and culture” [15]. The real-life contexts in which mobile systems are commonly used are often messy and variable, often involving a social context in which other people are present and different kinds of situations with various physical surroundings, such as on the bus or at home [16]. This variation and unpredictability of the context can constitute a challenge when designing and evaluating mobile systems, in addition to the methodological challenges of conducting trials in-the-wild [17], which are particularly applicable to mHealth technologies. Given the prevalence of mobile phones and the confidence the owners have in using them, it seems that usability evaluations of mobile technology are ideal to carry out as field trials, and there is a call for in-the-wild research studies of the use of mobile technology [15]. Yet, there are obstacles to conduct usability evaluations as field trials, for example, the potential difficulties in recreating the intended use situation, combining traditional usability evaluation techniques such as observation and walk-through, and controlling and accounting for all the variables in the environment [16].

As mentioned above, usability evaluation is tightly connected with interaction design. The affordances of mobile devices pose challenges that are particular to designing mobile apps. Compared with desktop computers, mobile devices have several limitations, specifically related to the mobile context in which they are used, such as connectivity, small screen size, different display resolutions, limited processing capability, power, and methods of data entry [18]. On the contrary, mobile devices offer new interaction modalities, such as gestures and movement, location, scan-and-tilt [19], point-of-view and head tracking [20], multitouch and video projection [21], context and proximity sensing, auditory input, and combinations of these features [22]. The proliferation of mobile devices among people all over the world and the many opportunities they provide for creating novel interaction forms raise the question of whether these features are being used in the design of mHealth apps or whether mobile platforms are mainly considered as a convenient way of delivering information.

### Objectives

Given the background described above, the goal of our systematic review was to increase the understanding of how usability is being addressed and measured in mobile interventions focusing on mental health problems, where the interventions are made available using mobile devices. We also examined how participants were recruited and which user representatives were involved in the 42 studies from the literature. The following research questions guided the review:

1. What is the approach to users taken in the studies?
2. What are the objectives and outcomes of the studies?
3. What are the characteristics of the mobile apps in the interventions? 
4. Why are apps being developed for mobile platforms?
5. Which research methods and techniques are being used to conduct usability evaluation in the studies?

## Methods

### Study Design

The scope of our review includes how designers approach usability evaluation of new tools and those that are still being developed, that is, their overall evaluation strategies or research approaches to usability evaluation and the concrete methods and usability scales that are being used. We examined how participants are recruited into studies that conduct evaluations of apps, for example, whether the participants are patients diagnosed with a mental illness or mental health professionals and which user representatives are involved. As an aspect of the research approach, usability can be incorporated in the design process and in assessing a developed tool, which can be in the form of co-design or user-centered design, in which future users take part and influence the design process. We looked at the maturity levels of the systems included in the review and the stated purpose of the evaluation. We also reviewed the articles for which mental health issue systems are being designed. Finally, this review assesses the different approaches to mobility as presented in the selected research articles. The reasons for deploying a mental health intervention on mobile devices vary, but in this study, we are primarily interested in why it is a popular platform for deploying mental health interventions.

### Information Sources and Search Strategies

A systematic search covering the scientific literature was performed in the medical databases EMBASE, CINAHL, PsycINFO, and PubMed and the wide-ranging scientific database Web of Science. The search was limited to papers published between January 2001 and the end of December 2018. The results were compared and consolidated after each step. The databases were chosen to ensure that all relevant articles could be included in the review study. Search terms were based on a combination of the following keywords: usability, evaluation, assessment, measure, test, testing, heuristics, mental health, mental illness, mental disorder, psychiatric illness, mobile health, M-health, e-health, internet, cCBT (computerized cognitive behavioral therapy), and computerized CBT. The keywords were combined using the Boolean operators OR and AND. The search was customized for each selected database in accordance with their filtering specifications.

### Database Searching Process

This review focused on the 4 areas of usability, evaluation methods, mental health, and mobile digital interventions, and accessed relevant articles throughout the 4 steps. Therefore, the search keywords used included words related to these areas. The first keyword was “usability,” which is the main focus of this review. Second, the keywords related to usability evaluation methods were added to identify the applied assessment methods of articles (eg, evaluation OR assessment OR measure OR test OR testing OR heuristics). Third, the results were refined to include the keywords related to the mental health domain (eg, mental health OR mental illness OR mental disorder OR psychiatric illness). Fourth, search keywords addressing mobile digital intervention in mental health were added to limit the search results and access more relevant articles.

### Selection of Studies

The Preferred Reporting Items for Systematic Review and Meta-Analysis statement was used for the reporting of the systematic review [23]. A total of 5 databases were searched systematically using predetermined keywords. The reference lists of the included articles were also searched for additional relevant articles. After removing duplicates, a set of inclusion and exclusion criteria were formulated to evaluate and identify the most relevant articles.

We included those studies in which the articles met the following inclusion criteria (1) focusing on usability evaluation of a mobile digital mental health intervention and (2) providing empirical evidence with regard to the usability evaluation outcomes of digital mental health interventions. We also excluded the studies that met at least one of these exclusion criteria: (1) not written in English; (2) published before 2001 or after December 2018; (3) not having a full text or published in the form of a conference paper or an abstract; (4) designed as nonempirical research (eg, opinion papers, reviews, editorials, and letters); (5) study protocol; (6) dealing with usability evaluation in domains that do not include mobile digital mental health; and (7) having limited mobile use to SMS, as a Web browsing platform, or purely as a sensor.

The database searches were performed by 2 of the authors independently in a double-blind process. After identifying relevant articles through the electronic database search, 65 duplicate articles were removed, and 234 unique articles remained. In the screening step, the resulting list of 234 articles were reviewed independently by the same 2 authors according to the inclusion and exclusion criteria by considering the title, keywords, and abstract, and all 59 eligible studies were retrieved. To assess the eligibility of the remaining articles, the full texts were evaluated, provided the information given in the abstract was sufficient to decide on the relevance of the article.

**Figure 1:**
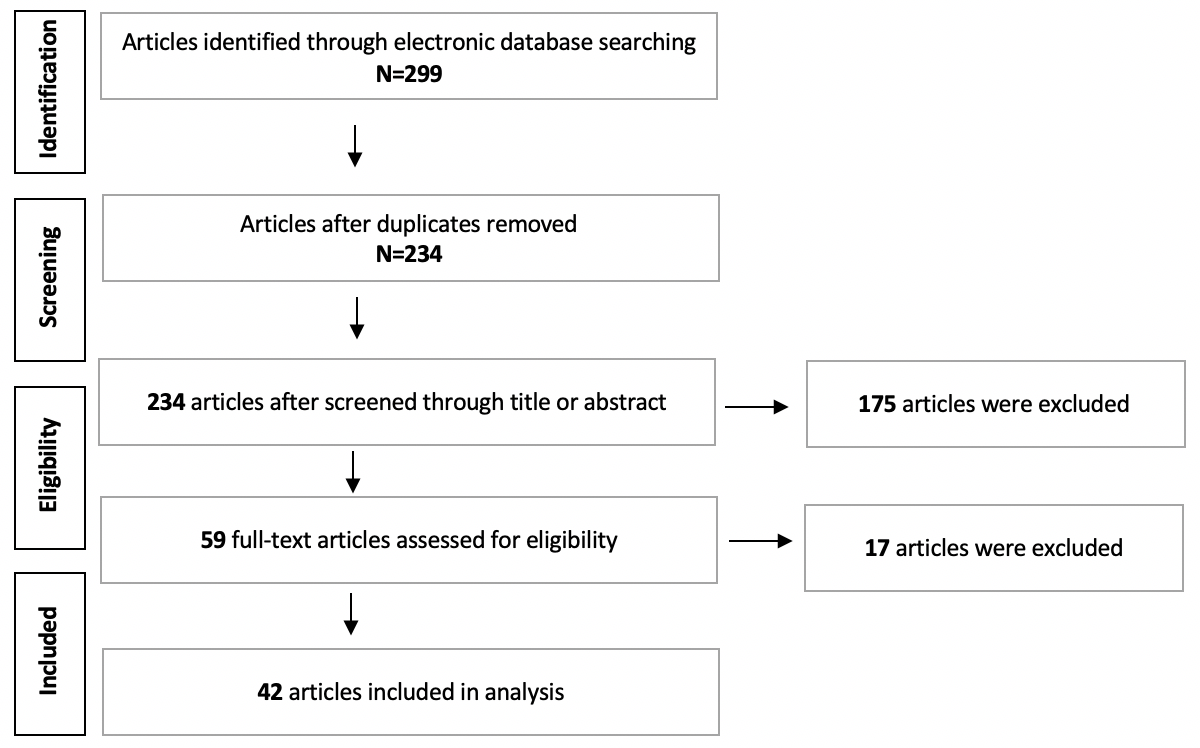
Preferred Reporting Items for Systematic Review and Meta-Analysis (PRISMA) flow diagram of article inclusion.

The full texts of all identified articles were assessed independently by the same authors. Articles upon which both authors agreed were included. Any discrepancies between the authors regarding the selection of the articles were discussed, and a consensus was reached on all reviewed articles in a joint session. In total, 17 articles were excluded in this round, and the selection process led to the inclusion of 42 articles in this review as shown in Figure 1. The main method to resolve discrepancies was to review the full text paper with regards to IC2: whether the paper described a usability study including empirical evidence.

### Defining the Evaluation Criteria Used in the Study

In accordance with the research questions, the evaluation criteria were grouped according to a set of 4 themes: (1) approach to *the user*, (2) *objectives and outcomes* of the study, (3) *research methods* and techniques to conduct usability evaluation, and (4) information about the *mHealth interventions*. A summary of the themes, evaluation criteria, and their values are given in Table 1.

The theme *approach to the users* contains information that is descriptive of the participants. The mental health problem addressed in the study refers to the stated diagnosis or mental health symptoms that the intervention is directed at. These can be diagnosis-based, symptom-based, and some interventions are also more general in nature, or about *wellness* or providing *access to information*. The *sample size* refers to how many participants took part in the evaluation, and we included a description of their role, that is, being patients, experts, health professionals, relatives, and so on. In *target demographics*, we reviewed whether there were any particular social strata that were addressed through the intervention in addition to the diagnosis, such as gender, age group, and culture.

**Table 1:** Themes, evaluation criteria, and main subvalues and categories used in the study.

Theme	Evaluation criteria	Main subvalues and categories
Approach to the users	Type of mental health problem/diagnosis, sample size, target demographics	—^a^
Study objectives and outcomes	Purpose of the study, outcome of the study	Outcome: User reception, tool improvement, design recommendations, design themes, value in exploration, medical outcomes (positive, negative, and neutral), research improvement and app/tool
Methods and techniques	Research methods, usability evaluation techniques, and purpose of the usability evaluation	Research methods: Trial, user-centered design, mixed methods study, and participatory design; Evaluation methods: Interview (type), think-aloud, questionnaire (type), field study (natural environment), app use data, co-operative design, verbal probing, observation, scenario-based tasks, focus-group, panel review, video recording, logging/diaries, task-based evaluation, wireframing/sketching, personas; Purpose: Formative, summative
Mental health intervention	Maturity level of the mobile system and approach to mobility	Approach category: Device affordances, availability of mobile technology, contextual support, novelty of mobile research, popularity, user maturity, and privacy of mobile use

^a^The theme has no subvalue or category.

The theme *objectives and outcomes* describe the author’s stated purpose of the article. The meaning of purpose was considered self-explanatory. We found statements of objective in the abstract section in most of the papers, where the articles had a subheading in the abstract where this was described explicitly. We also reviewed the introduction and methods sections, in which the objectives of the studies were explained. Outcome descriptions were grouped and categorized according to their meaning. The outcomes refer to conclusions about the main contributions of the study, as described by the authors. The outcomes were categorized using the terms user reception, tool improvement, design themes, value in exploration, medical outcomes (positive, negative, and neutral), research improvement, and app/tool. User reception refers to conclusions about how the technology was received and perceived by the participants in the study. Tool improvement means the conclusions about how the technology was improved are based on the feedback from the users. Design themes are observations that are sufficiently general to be of value to other designers and developers of mobile mental health technologies. Value in exploration means that the authors found that taking an explorative approach to evaluation gave significant knowledge in return for the study. Medical outcomes are conclusions concerning the medical effects on the participants of the study. Research improvement are findings that increase quality of future mHealth usability evaluations. Finally, app/tool refers to conclusions about how the technology was accomplished, usually based on user feedback.

*Methods and techniques* describe the methodological aspects of the studies. Research methods refer to the overall research strategy employed in the study that the article describes. We decided on the following main types of research methods: trial, user-centered design, mixed methods study, and participatory design. A trial is about determining the effects of an intervention. A criterion for a trial was that the participants used the technology independently for a period either in their natural environment or elsewhere. If the trial took place in the user’s natural environment, this was specified in the usability evaluation column. Mixed methods studies refer to a set of qualitative and/or quantitative techniques being used to study the intervention but not in the sense of a trial. User-centered design methods refer to studies where potential users or representatives of users have taken part in the design process, for example, in the form of co-design or participatory design. User-centered design methods also usually entail an iterative design and evaluation process. The purpose of the evaluation describes whether intervention technology redesign based on user input took place in the study, described as formative if so and summative where the focus of the study was on describing the usability evaluation results. Usability evaluation refers to the data collection techniques that were used in the study, that is, interviews, observation, and questionnaires, including standardized usability measures, such as the System Usability Scale (SUS) or their adaptations.

The theme *mHealth intervention* refers to information about digital intervention. By reviewing the *maturity of the technology*, we wanted to see if there were discerning trends in how early in development usability evaluations were carried out. For maturity, we distinguished between the main categories of sketch, prototype, matured, and released version of technology. In *approach to mobility*, we were interested in if and how the authors argued for the choice to use mobile technology for their intervention. The main categories for these were device affordances, availability of mobile technology, contextual support, novelty of mobile research, popularity, user maturity, and privacy of mobile use. Interventions that employ mobile devices because of specific affordances (sensors, communication, etc) use aspects of mobile devices that are difficult or less practical to replicate using other devices. Availability refers to the notion that mobile devices are available to the user most of the time. Popularity means that mobile devices are currently in use by most people for most age groups. User maturity refers to the idea that people have become technologically proficient in mobile phone use. Privacy is related to the notion that people regard, for example, finding information on their mobile phone as more private than visiting a mental health professional.

## Results

### Approach to the Users

A total of 29 of the studies conducted a usability evaluation of a mobile mental health program with either patients or patient families with parent-child dyads, 11 with health care providers, such as clinicians, caregivers, nurses, therapists, care managers, and health professionals, and 8 with healthy users. Of the studies gathering feedback from users, 1 study recruited users with a history of trauma, encouraged users with lived experience of mental health and substance use to participate, and evaluated usability with users who were offspring of patients with dementia, and another recruited parents of children with neurodevelopmental disabilities. To conduct a more detailed analysis, some of the studies performed the usability evaluation of a mobile mental health program with different user groups. A total of 6 studies obtained usability feedback from both patients and health care providers. The categories healthy users and patients, healthy users and health care providers, healthy users and experts, health care providers and experts, patients and practitioners, patients and teachers of dyslexia, health care providers, patients and researchers on health domain, and health care providers, patients and healthy users occurred once (see Table 2).

**Table 2:** Types of users recruited by the reviewed studies.

User type	Study
Patients	Auger et al, 2014 [24]; Barrio et al, 2017 [25]; Bauer et al, 2018 [26]; Ben-Zeev et al, 2013 [27]; Ben-Zeev et al, 2014 [28]; Boman and Bartfai, 2015 [29]; Boyd et al, 2017 [30]; Corden et al, 2016 [31]; Deady et al, 2018 [32]; Dulin et al, 2014 [33]; Fuller-Tyszkiewicz et al, 2018 [34]; Henry et al, 2017 [35]; Huguet et al, 2015 [36]; Juengst et al, 2015 [37]; Kobak et al, 2015 [38]; Latif et al, 2015 [39]; Macias et al, 2015 [40]; Meiland et al, 2012 [41]; Mistler et al, 2017 [42]; Morland et al, 2016 [43]; Nicholson et al, 2018 [44]; Nitsch et al, 2016 [45]; Palmier-Claus et al, 2013 [46]; Prada et al, 2017 [47]; Rizvi et al, 2016 [48]; Rohatagi et al, 2016 [49]; Ruggiero et al, 2015 [50]; Sze et al, 2015 [51]; Whiteman et al, 2017 [52]
Health care providers	Bauer et al, 2018 [26]; Boman and Bartfai, 2015 [29]; Fuller-Tyszkiewicz et al, 2018 [34]; Kobak et al, 2015 [38]; Meiland et al, 2012 [41]; Ospina-Pinillos et al, 2018 [53]; Rohatagi et al, 2016 [49]; Ruggiero et al, 2015 [50]; Sands et al 2016, [54]; Villalobos et al, 2017 [55]; Wood et al, 2017 [56]
Healthy users	Boyd et al, 2017 [30]; Carey et al, 2016 [57]; Connelly et al, 2016 [58]; de Korte et al, 2018 [59]; Garcia et al, 2017 [60]; Kizakevich et al, 2018 [61]; Ospina-Pinillos et al, 2018 [53]; Rohatagi et al, 2016 [49]
Users with a mental health history	Price et al, 2016 [62]
Users with lived experience of mental health and substance use	VanHeerwaarden et al, 2018 [63]
Users who were offspring of patients with a mental health illness	van Osch et al, 2015 [64]
Users who were parents of children with a mental health	Jiam et al, 2017 [65]
Researchers on health domain	Fuller-Tyszkiewicz et al, 2018 [34]
Teachers of dyslexia	Latif et al, 2015 [39]
Practitioners	Ben-Zeev et al, 2013 [27]
Experts	de Korte et al, 2018 [59]; Sands et al, 2016 [54]

The total sample size at baseline (regardless of the number of groups) ranged from 5 [24,43] to 3977 [60]. A total of 3 studies reported targeting only females [45,47,58], whereas 1 study gathered data only from male patients [29] and male users [61]. There was an equal gender distribution in 4 studies [25,40,52,55]. One study recruited the same number of males and females in stage 1, but all males in stage 2 [32], and another study included 1 group of users (young people), and not the other group (youth health professional) [53]. Gender information was not reported in 3 studies about health care providers [29,49,50], in 2 about users [43,61], in 1 about teachers of dyslexia [39], in 1 about practitioners [27], in 1 about experts and health care providers [54], and in another about users and health care providers [26]. A total of 8 studies reported only the age range of the participants [36,40,44,45,55,57,63,65], 6 provided only the mean age [25,28,31,34,46,62], and 2 did not provide this information [26,60]. Although some of the studies gathered usability feedback from different user groups, such as both patients and health care providers or patients and healthy users, a considerable number of the studies (n=12) did not present the same level of detailed information about all participants’ demographics for each user group, such as the mean age, age range, and gender [27,29,30,35,49-51,53,58,61].

A significant number of the included studies addressed generic mental health issues, such as well-being, mindfulness, and goal achievement, followed by depression, schizophrenia, alcohol use disorder, bipolar disorder, cognitive impairment, eating disorder and serious mental illness, borderline personality disorder, dementia, medical adherence, and posttraumatic stress disorder. The full list of mental health problems is presented in Table 3.

**Table 3:** Mental health problems addressed in the studies.

Mental health problem	Study
A history of violence	Mistler et al, 2017 [42]
Alcohol dependence and misuse	Barrio et al, 2017 [25]; Kizakevich et al, 2018 [61]; Dulin et al, 2014 [33]
Anger	Morland et al, 2016 [43]
Bipolar disorder	Bauer et al, 2018 [26]; Macias et al, 2015 [40]; Mistler et al, 2017 [42]
Borderline personality disorder	Prada et al, 2017 [47]; Rizvi et al, 2016 [48]
Burnout	Wood et al, 2017 [56]
Cognitive impairment	Boman and Bartfai, 2015 [29]; Boyd et al, 2017 [30]; Auger et al, 2014 [24]
Dementia	Meiland et al, 2012 [41]; van Osch et al, 2015 [64]
Depression	Corden et al, 2016 [31]; Deady et al, 2018 [32]; Fuller-Tyszkiewicz et al, 2018 [34]; Kobak et al, 2015 [38]; Macias et al, 2015 [40]
Eating disorders	Connelly et al, 2016 [58]; Nitsch et al, 2016 [45]; Sze et al, 2015 [51]
Dyslexia	Latif et al, 2015 [39]
Generic (communication access, assessment, contentment, well-being, goal achievement, and mindfulness)	Carey et al, 2016 [57]; de Korte et al, 2018 [59]; Garcia et al, 2017 [60]; Ospina-Pinillos et al, 2018 [53]; Ruggiero et al, 2015 [50]; Sands et al, 2016 [54]; VanHeerwaarden et al, 2018 [63]; Villalobos et al, 2017 [55]
Headache	Huguet et al, 2015 [36]
Medication adherence	Corden et al, 2016 [31]; Rohatagi et al, 2016 [49]
Neurodevelopmental disabilities	Jiam et al, 2017 [65]
Posttraumatic stress disorder	Bauer et al, 2018 [26]; Price et al, 2016 [62]
Psychosis	Palmier-Claus et al, 2013 [46]
Schizoaffective disorder	Mistler et al, 2017 [42]
Schizophrenia	Ben-Zeev et al, 2013 [27], 2014 [28]; Macias et al, 2015 [40]; Mistler et al, 2017 [42]; Palmier-Claus et al, 2013 [46]
Serious mental illness	Whiteman et al, 2017 [52]; Nicholson et al, 2018 [44]; Rohatagi et al, 2016 [49]
Sleep problems	Kizakevich et al, 2018 [61]
Stress	Kizakevich et al, 2018 [61]
Tinnitus	Henry et al, 2017 [35]
Traumatic brain injury	Juengst et al, 2015 [37]

### Objectives and Outcomes

Across the studies, the reported primary purposes differed considerably. Half of the studies emphasized usability evaluation [24,25,29-64], 10 focused on feasibility [28,31,32,36-38,42,44,48,51] and acceptability [28,32,40,42,47,48,51,56,60], and for 5, effectiveness [32,33,38,48,56] was the main objective. Some of the studies had the purpose of concentrating on patients attitudes, such as satisfaction [25,38], perception [46], openness [47], motivation [64], opinions [59], and adherence to the use of a mobile mental health app [49], whereas others addressed mobile apps, for example, system usage [33,44], app optimization [63,64], validity of a mHealth system [37], efficacy [28], usefulness [44], perceived quality [60], content validity [54], significant features in content [61], safety [49], psychometric properties [36], and health assessment quality [61].

Numerous studies described the process of design [53,58], development [27,30,32,35,36,49,50,53,65], and adaptation [55] of a mobile mental health app or platform, whereas a few aimed to demonstrate the value of usability research [45] and benefits of mobile technologies in providing a learning platform [39] or examined how to incorporate mobile technologies to support delivery of a mental health service [26]. Only 2 studies targeted to test an intervention [35] and improve the treatment of depression [31].

The outcomes of almost all of the included studies, except one, were user reception, followed by medical outcome (positive), tool improvement, app/tool, design recommendations, design themes, medical outcome (potential), medical outcome (neutral), and product and implementation issues. Outcomes that occurred once were value in exploration, research improvement, medical outcome (indirectly), design principles, and evaluation knowledge. Details are given in Table 4.

**Table 4:** Outcomes of the included studies.

Outcome	Study
User reception	Auger et al, 2014 [24]; Barrio et al, 2017 [25]; Bauer et al, 2018 [26]; Ben-Zeev et al, 2013 [27], 2014 [28]; Boman and Bartfai, 2015 [29]; Boyd et al, 2017 [30]; Carey et al, 2016 [57]; Connelly et al, 2016 [58]; Corden et al, 2016 [31]; de Korte et al, 2018 [59]; Deady et al, 2018 [32]; Dulin et al, 2014 [33]; Fuller-Tyszkiewicz et al, 2018 [34]; Garcia et al, 2017 [60]; Henry et al, 2017 [35]; Huguet et al, 2015 [36]; Jiam et al, 2017 [65]; Juengst et al, 2015 [37]; Kizakevich et al, 2018 [61]; Kobak et al, 2015 [38]; Latif et al, 2015 [39]; Macias et al, 2015 [40]; Meiland et al, 2012 [41]; Mistler et al, 2017 [42]; Morland et al, 2016 [43]; Nicholson et al, 2018 [44]; Nitsch et al, 2016 [45]; Ospina-Pinillos et al, 2018 [53]; Palmier-Claus et al, 2013 [46]; Prada et al, 2017 [47]; Price et al, 2016 [62]; Rizvi et al, 2016 [48]; Rohatagi et al, 2016 [49]; Sands et al, 2016 [54]; Sze et al, 2015 [51]; van Osch et al, 2015 [64]; VanHeerwaarden et al, 2018 [63]; Villalobos et al, 2017 [55]; Whiteman et al, 2017 [52]; Wood et al, 2017 [56]
Medical outcome (positive)	Ben-Zeev et al, 2014 [28]; Carey et al, 2016 [57]; Corden et al, 2016 [31]; Deady et al, 2018 [32]; Dulin et al, 2014 [33]; Garcia et al, 2017 [60]; Huguet et al, 2015 [36]; Juengst et al, 2015 [37]; Kobak et al, 2015 [38]; Macias et al, 2015 [40]; Mistler et al, 2017 [42]; Prada et al, 2017 [47]; Rizvi et al, 2016 [48]; Sze et al, 2015 [51]; Wood et al, 2017 [56]
Tool improvement	Connelly et al, 2016 [58]; Henry et al, 2017 [35]; Jiam et al, 2017 [65]; Meiland et al, 2012 [41]; Nitsch et al, 2016 [45]; Ruggiero et al, 2015 [50]; Sands et al, 2016 [54]; van Osch et al, 2015 [64]; Whiteman et al, 2017 [52]
App/tool	Ben-Zeev et al, 2013 [27]; Connelly et al, 2016 [58]; Deady et al, 2018 [32]; Henry et al, 2017 [35]; Latif et al, 2015 [39]; Ospina-Pinillos et al, 2018 [53]; Ruggiero et al, 2015 [50]; VanHeerwaarden et al, 2018 [63]
Design recommendations	Dulin et al, 2014 [33]; Fuller-Tyszkiewicz et al, 2018 [34]; Garcia et al, 2017 [60]; Juengst et al, 2015 [37]; Ospina-Pinillos et al, 2018 [53]; Price et al, 2016 [62]
Design themes	Auger et al, 2014 [24]; Connelly et al, 2016 [58]; Nitsch et al, 2016 [45]
Medical outcome (potential)	Latif et al, 2015 [39]; Ruggiero et al, 2015 [50]; Whiteman et al, 2017 [52]
Medical outcome (neutral)	Kizakevich et al, 2018 [61]; Meiland et al, 2012 [41]
Product	Henry et al, 2017 [35]; Ruggiero et al, 2015 [50]
Implementation issues	Boman and Bartfai, 2015 [29]; Palmier-Claus et al, 2013 [46]
Value in exploration	Villalobos et al, 2017 [55]
Research improvement	Macias et al, 2015 [40]
Medical outcome (indirectly)	Boman and Bartfai, 2015 [29]
Design principles	Bauer et al, 2018 [26]
Evaluation knowledge	de Korte et al, 2018 [59]

### Characteristics of Mobile Health Interventions

**Figure 2:**
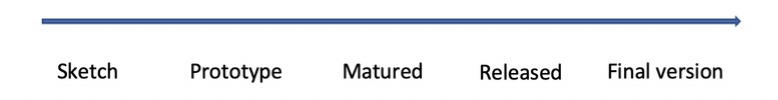
Mobile health technology maturity scale.

The maturity level of the mobile systems that were reviewed were placed on a continuum from sketch to final product (Figure 2). A sketch-to-prototype means that the study described the development of the app in the form of co-design and that gleaning feedback and worldviews of the users were the focus of the study. A prototype is the minimally working version of an app with functionality that the user can test. A matured version is an app that has been tested by users and redesigned/amended in some way. A released version refers to the app being downloadable from an app store or elsewhere, and the final version is self-explanatory.

The most common maturity level of technology in the review was divided among 13 studies of matured version of technology, 8 studies of released version technology, 9 prototype, and 6 prototype-to-matured. There was only 1 paper that evaluated a final product, whereas 3 studies described the process from sketch to prototype. For 2 of the studies, no information about the maturity level was available (Table 5). For the categories sketch-to-prototype and prototype-to-matured, the studies were focused on describing a development process, where user feedback was used in a formative redesign of technology.

**Table 5:** Maturity levels of the mobile systems that were reviewed.

Maturity level	Study
Sketch	—^a^
Sketch-to-prototype	Ospina-Pinillos et al, 2018 [53]; Sands et al, 2016 [54]; Whiteman et al, 2017 [52]
Prototype	Ben-Zeev et al, 2013 [27]; Carey et al, 2016 [57]; Deady et al, 2018 [32]; Jiam et al, 2017 [65]; Latif et al, 2015 [39]; Nitsch et al, 2016 [45]; Price et al, 2016 [62]; Rohatagi et al, 2016 [49]; van Osch et al, 2015 [64]
Prototype-to-matured	Bauer et al, 2018 [26]; Connelly et al, 2016 [58]; Henry et al, 2017 [35]; Huguet et al, 2015 [36]; Meiland et al, 2012 [41]; Ruggiero et al, 2015 [50]
Matured	Barrio et al, 2017 [25]; Ben-Zeev et al, 2014 [28]; Corden et al, 2016 [31]; de Korte et al, 2018 [59]; Dulin et al, 2014 [33]; Fuller-Tyszkiewicz et al, 2018 [34]; Garcia et al, 2017 [60]; Juengst et al, 2015 [37]; Macias et al, 2015 [40]; Nicholson et al, 2018 [44]; Palmier-Claus et al, 2013 [46]; Sze et al, 2015 [51]; VanHeerwaarden et al, 2018 [63]
Released version	Auger et al, 2014 [24]; Boyd et al, 2017 [30]; Kizakevich et al, 2018 [61]; Mistler et al, 2017 [42]; Morland et al, 2016 [43]; Prada et al, 2017 [47]; Rizvi et al, 2016 [48]; Wood et al, 2017 [56]
Final version	Boman and Bartfai, 2015 [29]
No information	Kobak et al, 2015 [38]; Villalobos et al, 2017 [55]

^a^Not applicable.

We reviewed the articles concerning how the authors argued for the use of mobile devices, and which and how mobile device affordances were used to make a tool. A summary of approaches to mobility results is given in Table 6. The availability of mobile devices was the most commonly cited reason to develop mHealth tools, which was found in 21 of the articles. The current popularity of mobile devices was mentioned in 16 of the studies, whereas 14 studies referred to or used affordances that are difficult to replicate on nonmobile devices, such as sensors. For 9 of the articles, this affordance was the potential for facilitating communication. A total of 8 papers referred to the novelty of mobile research, that is, it is worth exploring mHealth because it is relatively new and unexplored territory. A total of 8 articles referred to user maturity, meaning that their intended users were proficient in the use of mobile devices, whereas 5 papers mentioned the potential privacy of mobile use. A total of 4 articles pointed to the successful use of mobile technology in previous mHealth research, whereas 2 focused on how mHealth technologies could give the user control over their mental health problems. For 3 of the papers, no information was available. Overall, 1 paper each referred to how mHealth technology could augment existing practices, how it could increase cost effectiveness, and how it could support scalable solutions.

**Table 6:** Approaches to mobility.

Mobility approach	Study
Availability	Barrio et al, 2017 [25]; Ben-Zeev et al, 2014 [28]; Carey et al, 2016 [57]; Connelly et al, 2016 [58]; de Korte et al, 2018 [59]; Deady et al, 2018 [32]; Garcia et al, 2017 [60]; Henry et al, 2017 [35]; Huguet et al, 2015 [36]; Jiam et al, 2017 [65]; Juengst et al, 2015 [37]; Latif et al, 2015 [39]; Morland et al, 2016 [43]; Nicholson et al, 2018 [44]; Palmier-Claus et al, 2013 [46]; Prada et al, 2017 [47]; Price et al, 2016 [62]; Rizvi et al, 2016 [48]; Sands et al, 2016 [54]; Whiteman et al, 2017 [52]; Wood et al, 2017 [56]
Popularity	Bauer et al, 2018 [26]; Ben-Zeev et al, 2013 [27]; Ben-Zeev et al, 2014 [28]; de Korte et al, 2018 [59]; Deady et al, 2018 [32]; Dulin et al, 2014 [33]; Garcia et al, 2017 [60]; Kizakevich et al, 2018 [61]; Kobak et al, 2015 [38]; Mistler et al, 2017 [42]; Nicholson et al, 2018 [44]; Ospina-Pinillos et al, 2018 [53]; Prada et al, 2017 [47]; Price et al, 2016 [62]; Rizvi et al, 2016 [48]; Whiteman et al, 2017 [52]
Device affordances	Auger et al, 2014 [24]; Barrio et al, 2017 [25]; Boman and Bartfai, 2015 [29]; Corden et al, 2016 [31]; de Korte et al, 2018 [59]; Kizakevich et al, 2018 [61]; Latif et al, 2015 [39]; Mistler et al, 2017 [42]; Morland et al, 2016 [43]; Palmier-Claus et al, 2013 [46]; Price et al, 2016 [62]; Rohatagi et al, 2016 [49]; Ruggiero et al, 2015 [50]; van Osch et al, 2015 [64]
Communication affordance	Barrio et al, 2017 [25]; Bauer et al, 2018 [26]; Boman and Bartfai, 2015 [29]; Carey et al, 2016 [57]; Jiam et al, 2017 [65]; Kobak et al, 2015 [38]; Nitsch et al, [45]; Price et al, 2016 [62]; van Osch et al, 2015 [64]
Novelty of mobile research	Auger et al, 2014 [24]; Barrio et al, 2017 [25]; Bauer et al, 2018 [26]; de Korte et al, 2018 [59]; Juengst et al, 2015 [37]; Macias et al, 2015 [40]; Sze et al, 2015 [51]; Villalobos et al, 2017 [55]
User maturity	Ben-Zeev et al, 2013 [27]; Fuller-Tyszkiewicz et al, 2018 [34]; Jiam et al, 2017 [65]; Juengst et al, 2015 [37]; Nicholson et al, 2018 [44]; Rohatagi et al, 2016 [49]; Sze et al, 2015 [51]; Whiteman et al, 2017 [52]
Privacy of mobile use	Dulin et al, 2014 [33]; Kizakevich et al, 2018 [61]; Macias et al, 2015 [40]; Nicholson et al, 2018 [44]; Ospina-Pinillos et al, 2018 [53]
Scientific evidence of a positive effect	Deady et al, 2018 [32]; Fuller-Tyszkiewicz et al, 2018 [34]; Morland et al, 2016 [43]; Wood et al, 2017 [56]
Contextual support	Ben-Zeev et al, 2013 [27]; Macias et al, 2015 [40]; Villalobos et al, 2017 [55]
No information	Boyd et al, 2017 [30]; Meiland et al, 2012 [41]; VanHeerwaarden et al, 2018 [63]
User control	de Korte et al, 2018 [59]; Palmier-Claus et al, 2013 [46]
Augment existing practice	Sands et al, 2016 [54]
Cost effectiveness	de Korte et al, 2018 [59]
Scalability	Ruggiero et al, 2015 [50]

### Research Methods and Techniques of the Studies

Regarding the purpose of the usability evaluation, 31 of the included studies carried out a summative evaluation [24,25,27,64], whereas 11 undertook a formative evaluation [26,32,35-65]. A total of 3 studies carried out both summative and formative evaluations in separate phases [27,50,58]. A total of 32 studies were described as trials [25-27,30,31,33-35,38-65], whereas 12 used the method of user-centered design [24,26,28,32,35,39,41,50,52,58,64,65]. A total of 4 were mixed methods studies [29,44,62], and 3 were described as participatory design [36,53,66].

The most common data collection technique used was a questionnaire, either self-constructed, standard, or combinations of these. A total of 33 studies used questionnaires. In all, 31 studies were conducted as field studies in the natural environment of the participants, with the technology deployed in the everyday environment of the intended future user or the representatives of these users. A total of 23 studies made use of interviews. Less frequently used methods were observation, think-aloud, and the use of app-use generated data, task-based evaluation, and focus groups in order of frequency. The methods that were only referred to once in all the studies in the review were sensor data, co-operative design with both users and experts, verbal probing, user feedback, video recording, diaries, wireframing/sketching, personas, and journey mapping (see Table 7).

**Table 7:** Data collection techniques employed in the studies.

Outcome	Study
Questionnaire	Barrio et al, 2017 [25]; Bauer et al, 2018 [26]; Ben-Zeev et al, 2013 [27], 2014 [28]; Boman and Bartfai, 2015 [29]; Connelly et al, 2016 [58]; Corden et al, 2016 [31]; de Korte et al, 2018 [59]; Deady et al, 2018 [32]; Dulin et al, 2014 [33]; Fuller-Tyszkiewicz et al, 2018 [34]; Garcia et al, 2017 [60]; Huguet et al, 2015 [36]; Jiam et al, 2017 [65]; Juengst et al, 2015 [37]; Kizakevich et al, 2018 [61]; Kobak et al, 2015 [38]; Latif et al, 2015 [39]; Meiland et al, 2012 [41]; Mistler et al, 2017 [42]; Morland et al, 2016 [43]; Nicholson et al, 2018 [44]; Nitsch et al, 2016 [45]; Prada et al, 2017 [47]; Price et al, 2016 [62]; Rizvi et al, 2016 [48]; Rohatagi et al, 2016 [49]; Sze et al, 2015 [51]; van Osch et al, 2015 [64]; VanHeerwaarden et al, 2018 [63]; Villalobos et al, 2017 [55]; Whiteman et al, 2017 [52]; Wood et al, 2017 [56]
Field study	Auger et al, 2014 [24]; Barrio et al, 2017 [25]; Bauer et al, 2018 [26]; Ben-Zeev et al, 2013 [27]; Boman and Bartfai, 2015 [29]; Boyd et al, 2017 [30]; Carey et al, 2016 [57]; Corden et al, 2016 [31]; de Korte et al, 2018 [59]; Deady et al, 2018 [32]; Dulin et al, 2014 [33]; Fuller-Tyszkiewicz et al, 2018 [34]; Garcia et al, 2017 [60]; Henry et al, 2017 [35]; Jiam et al, 2017 [65]; Juengst et al, 2015 [37]; Kizakevich et al, 2018 [61]; Kobak et al, 2015 [38]; Macias et al, 2015 [40]; Meiland et al, 2012 [41]; Mistler et al, 2017 [42]; Morland et al, 2016 [43]; Nicholson et al, 2018 [44]; Palmier-Claus et al, 2013 [46]; Prada et al, 2017 [47]; Rizvi et al, 2016 [48]; Rohatagi et al, 2016 [49]; Sands et al, 2016 [54]; Sze et al, 2015 [51]; Villalobos et al, 2017 [55]; Wood et al, 2017 [56]
Interview	Auger et al, 2014 [24]; Boman and Bartfai, 2015 [29]; Carey et al, 2016 [57]; Connelly et al, 2016 [58]; Corden et al, 2016 [31]; de Korte et al, 2018 [59]; Dulin et al, 2014 [33]; Fuller-Tyszkiewicz et al, 2018 [34]; Huguet et al, 2015 [36]; Kizakevich et al, 2018 [61]; Meiland et al, 2012 [41]; Mistler et al, 2017 [42]; Morland et al, 2016 [43]; Nicholson et al, 2018 [44]; Nitsch et al, 2016 [45]; Ospina-Pinillos et al, 2018 [53]; Palmier-Claus et al, 2013 [46]; Price et al, 2016 [62]; Rohatagi et al, 2016 [49]; Ruggiero et al, 2015 [50]; Sands et al, 2016 [54]; van Osch et al, 2015 [64]; Villalobos et al, 2017 [55]
Observation	Auger et al, 2014 [24]; Boyd et al, 2017 [30]; Henry et al, 2017 [35]; Meiland et al, 2012 [41]; Ospina-Pinillos et al, 2018 [53]; Price et al, 2016 [62]; van Osch et al, 2015 [64]
Think-aloud	Ben-Zeev et al, 2014 [28]; Latif et al, 2015 [39]; Nitsch et al, 2016 [45]; Ospina-Pinillos et al, 2018 [53]; van Osch et al, 2015 [64]; Whiteman et al, 2017 [52]
App-use generated data	Dulin et al, 2014 [33]; Garcia et al, 2017 [60]; Macias et al, 2015 [40]; Nicholson et al, 2018 [44]
Task-based evaluation	Ben-Zeev et al, 2013 [27]; Henry et al, 2017 [35]; Ospina-Pinillos et al, 2018 [53]; van Osch et al, 2015 [64]
Focus group	Connelly et al, 2016 [58]; Garcia et al, 2017 [60]; Ruggiero et al, 2015 [50]
Sensor data	Garcia et al, 2017 [60]
Cooperative design	Whiteman et al, 2017 [52]
Verbal probing	Whiteman et al, 2017 [52]
User feedback	Sands et al, 2016 [54]
Video recording	Price et al, 2016 [62]
Diary	Meiland et al, 2012 [41]
Wireframing/sketching	Ospina-Pinillos et al, 2018 [53]
Personas	VanHeerwaarden et al, 2018 [63]
Journey mapping	VanHeerwaarden et al, 2018 [63]

Table 8 lists the type of evaluations undertaken in our review, either formative or summative, according to the evaluator having a medical or computer science background obtained from author affiliations and biographies in the articles. A total of 3 papers reported both summative and formative usability evaluations as they reported on several phases of development. The most common occurrence was summative evaluations carried out by authors with a medical background. When computer scientists are involved in the usability evaluation, it is in collaboration with scientists with a medical background. There were no papers reporting a formative evaluation purely with authors who were computer scientists. We would expect the frequency of formative evaluations with computer scientists to be higher, as the goal of HCI research is improving technology, building on deep understanding of user perceptions and use patterns of technology. These observations could be explained by the table presenting the maturity of the technologies being evaluated and the evaluations being mostly concerned with matured and released versions of technologies.

**Table 8:** Author credentials category according to the evaluation type (N=42).

Type of evaluation	Medical credentials, studies (n)	Computer science credentials, studies (n)	Both, studies (n)
Formative evaluation	5	—^a^	9
Summative evaluation	17	3	11

^a^No study fulfills this criterion.

## Discussion

### Approach to the Users

According to the data yielded by the literature search, most of the studies conducted a usability evaluation of a mobile mental health program with diagnosed patients. It is possible to evaluate the usability of mHealth technologies with healthy users, but many of the studies in our review were simultaneous trials, with the goal of measuring health outcomes in addition to the effects of technology, which can explain the high number of studies that evaluated usability with patients. Although the evaluated programs were within the scope of the mental health domain, some of the studies recruited healthy users to measure usability and understand how to meet user expectations and needs. Studies gathering data from healthy users mainly followed a user-centered design approach, focusing on the development and evaluation process of a mobile app. For example, Connelly et al [58] developed a mobile app for low literacy to record unhealthy eating and weight control behaviors of Mexican American women. The authors completed the development process in 4 phases and conducted a final usability assessment. Similarly, Ospina-Pinillos et al [53] used participatory research methodologies to develop a mental health e-clinic for healthy young people across Australia. The authors included young people in all stages of the development process. It is interesting that the majority of the studies involved patients as it is much more difficult to recruit patients than healthy users, and for some diagnoses, such as dementia or schizophrenia, there may also be particular challenges in working with these patients to learn about the usability issues. In this respect, participation in the co-design and evaluation of technology to treat an illness also concerns the aspect of patient representation (eg, [67]); that is, those affected by a mental health problem should be able to influence the design of technology that is being made to treat and manage the problems.

Although most studies evaluated the usability of mobile mental health programs with a single user group, one-third enriched the usability data with different groups of potential users, such as patients, health care providers, healthy users, affected parents and children, and medical experts. For example, Boyd et al [30] involved both healthy users and patients, Ruggiero et al [50] included both health care providers and children, and Fuller-Tyszkiewicz et al [34] conducted a usability evaluation with health care providers, patients, and researchers in the health domain. The health care providers from whom the reviewed studies gathered data were clinicians, caregivers, nurses, therapists, care managers, or health professionals. When the goal of the mHealth technology is to change a medical practice, rather than improve health directly, it becomes important to involve other groups in the usability evaluations; for example, Boman and Bartfai [29] evaluated a physical robot as assistive technology for enhancing communication between patients and health care professionals. Each different user group or stakeholders may have different views on how useful it is and different types of use, which affects perceptions of usability. Fuller-Tyszkiewicz et al [34] tested the usability of a mobile app for depression with patients, mental health professionals, and researchers in the health domain. The WHO [5] identified health workers’ perceived barriers to using mHealth apps, and one of the main barriers was usability problems in the apps and problems with integrating the new tools with systems already in use. There are several barriers to changing existing work practices for health workers, and for mobile devices, health workers are concerned about the character limits on SMS messages, and limited/cumbersome note-taking capabilities [5]. However, health workers are interested in being involved in the design and evaluation of new technology [5].

Although the studies in this review aimed to test the usability of a mobile mental health program, only half emphasized usability evaluation as the main purpose. A total of 10 studies highlighted feasibility (“an assessment of the practicality of a proposed plan or method,” [68]) and acceptability (“the extent to which the assessment is experienced as probing yet unobtrusive,” [66]) in addition to the usability evaluation. A few of the studies addressed the components of usability, such as effectiveness, user satisfaction, and efficiency. A considerable number of studies described the process of software development life cycle including design, development or adaptation, whereas the majority of the studies carried out a summative evaluation. Only 11 studies engaged in a formative evaluation to gather feedback from users and improve the design as part of an iterative design process. This is in line with the finding of Nørgaard and Hornbæk [12], who reported that the data from the evaluation of prototypes was rarely used in interaction design for reasons such as the lack of action ability of the evaluation results and time pressure in the development process. Kjeldskov and Stage [16] also pointed out that it was easier to carry out formative evaluations early in the development process, whereas there were stronger obstacles to changing the designs later. Only 12 of the reviewed studies were in the sketch or prototype stages, whereas 39 were matured or released versions of technology. When evaluating the usability of a finished technology, the goal becomes to demonstrate the effectiveness and validate the design rather than improving it.

### Objectives and Outcomes

We found that many of the studies were heavily influenced by practices, ideas, and notions from randomized controlled trials, which is the standard practice for evidence building in medicine. Many of the studies set out to investigate the feasibility and acceptability of an mHealth app (eg, [28,32,42]), and usability measures were used as a step toward fulfilling this goal. Feasibility and acceptability are often the focus of the pilot phase in a randomized controlled trial for a new medical procedure or medication. The word *usability* was not included in the title of most of the studies, although usability evaluation was either the main goal or one of the goals. Some of the reviewed studies (eg, [28,30,32,51,54,57]) also examined feasibility and/or acceptability but used the terms usability and acceptability together. For some of the studies (eg, [32,38,61]), the goal was to measure effectiveness of a mobile app for a specific mental health problem. They mainly administered a usability questionnaire as a summative evaluation at the end of their field study or trial. Furthermore, there were studies measuring only simplicity of use (eg, [30]), ease of use, and usefulness (eg, [59]). There was a duality in the goals for and underlying assumptions of developing a digital tool to be usable, that is, an mHealth app, and attempting to improve a person’s health. In usability evaluations, the goal was to learn whether a tool is meaningful and how it could be improved, whereas in studies of health and medicine, the goal was to create a positive health effect for a person. When these goals are combined, the objective of the usability studies becomes explaining how the health effects are assisted or mediated by the mHealth app, and the usability evaluation evidence has a summative role.

Concerning the outcomes that the included studies presented, all but one referred to user reception as the main contribution of the study. This finding indicates that almost all the studies received positive feedback from their participants who found the evaluated tools useful. Positive medical outcome, tool improvement, app/tool, and design recommendations were other commonly reported results in the studies. These studies had conclusions about the positive medical effects of the evaluated tools on the participants and how the mobile mental health program was improved and accomplished based on user feedback and recommendations to researchers or practitioners on using similar technologies. We regard the reporting of medical outcomes in usability studies as a part of building evidence of the effectiveness of the mHealth technology. Some of the reviewed studies (eg, [58,64,65]) contained the development process of a mobile mental health app in detail, and the authors of these studies elaborated how a mobile app was improved following an iterative and incremental process based on user feedback. Whiteman et al [52] suggested that early involvement of users in the development process resulted in building a usable system. Similarly, Juengst et al [37] listed the lessons learned, such as the importance of a simple interface of a mobile app and effective communication between patients and health care providers. These results can be an important step on the journey from an idea for a mobile mental health intervention technology to its implementation and use in health care, for example, in warranting further research.

Reporting these kinds of outcomes can also be understood as an attempt to demonstrate that the mHealth technology works or does what it is intended to do, which is a common venture in HCI research. Klasnja et al [69] argued that electronic health technology evaluations should refrain from documenting behavioral changes, as behavioral change processes (1) are inherently complex, that is, subject to interconnected social, material-logistical, motivational, and circumstantial factors, and (2) need to have a very long–time frame to be of value. The problem of attrition or lack of sustained use has also been described as specific to mHealth apps (eg, [9]). Alternatively, the evaluation could focus directly on the underlying behavioral change strategies of the mHealth app to warrant or unwarrant further investigation of the medical efficacy of the app [69], for example, to determine whether a particular implementation of the strategy of self-monitoring the number of steps walked in a day actually increased the number of steps. Early in the design process of a technology for behavioral change, a deep understanding of the “how and why of the technology use by its target users should be a central goal for evaluations” according to Klasnja et al [69].

### Characteristics of the Mobile Health Interventions

Most of the reviewed studies evaluated matured versions of mobile mental health apps; therefore, the app had been previously tested by either users or patients and updated based on their feedback. This was followed by the released version, which refers to an app which is downloadable from a platform, such as Apple Store or Google Play, prototype version, which means the app has a high-fidelity version for users to test its usability and functionality, and prototype-to-matured version. Only 15 of the included studies were described as user-oriented design methods of either user-centered design or participatory design; however, most studies described their methods as trials. Studies using a user-oriented design method most often carried out a formative evaluation, whereas most of the studies describing their methods as a trial engaged in a summative evaluation.

The most commonly referred to reason for developing mHealth apps for mental health was the availability of mobile devices to users, their popularity, and how people in general became accustomed to using them for various purposes, for example, by pointing out how mobile technologies were in a “process of technological acceleration” [25], and a “mobile device explosion” [60] and that “smartphone users are with their phones for all but 2 hours every day” [57]. This way of supporting the development of apps for mobile use is arguably generic and transferable to other areas of mobile technology use, such as games and social media. Simultaneously, through the proliferation of digital technology into the private spheres of the users [14,15], mobiles have partly facilitated a shift in the design and use of technology, paving the way for fields of research, such as mHealth. A number of the studies approached mobility by regarding and making use of the affordances and characteristics specific to mobile devices, such as opportunities for sensing data about the user and their contexts [24] and facilitating communication [29]. A total of 5 papers mentioned the potential for mobiles to support privacy, which is in agreement with the WHO [5] report that found privacy in stigmatized health conditions as one of the feasibility enhancers for patients.

### Research Methods and Techniques of the Studies

User evaluation is an essential source of information to improve the usability of systems [70], aims to understand both positive and negative sides of an app, and provides valuable information in this regard [71]. To gather user feedback and evaluate the usability of mHealth apps, the most common data collection technique utilized in the studies was questionnaire, followed by field study, interview, observation, think-aloud, and app-use generated data. This result corroborates the ideas of Holzinger [72], who pointed out that among several usability assessment methods, questionnaire, think aloud, and observation were the most commonly used methods.

Accordingly, the SUS was the most common standard questionnaire used by the studies in the review. This scale, developed by Brooke [73], aims to measure perceived usability and is one of the well-established and popular scales in the HCI field. Some of the studies constructed their own questionnaires to evaluate the usability of mobile mental health programs based on available standard questionnaire(s). The SUS, the Usefulness, Satisfaction, and Ease of Use (USE) Questionnaire [74], and the Poststudy System Usability Questionnaire [75] were the most commonly used scales that studies used to create their own questionnaires. Almost half of the studies which used questionnaire as the main data collection technique either created a questionnaire based on one of those available or constructed their own questionnaires as available questionnaires did not entirely meet their needs in evaluating the usability of mobile mental health programs. Considering that the questionnaires were targeted toward desktop apps, our results unveil a need for a questionnaire focusing on testing the usability of mobile mental health apps. There can be good reason to tailor and adapt a standard questionnaire to a particular usability evaluation, but the authors are then expected to prove the reliability and validity of their questionnaire. Among the reviewed studies which either created new items or adapted a standard questionnaire, only Prada et al [47] provided a reliability score (Cronbach α=.88) of the questionnaire they developed.

### Implications and Recommendations for Future Mobile Health Research and Usability Evaluation

#### Involve Patients and Health Care Professionals in Mobile Health Development

As people access mHealth apps to improve their health, publishers of mHealth apps have a responsibility to ensure the medical quality of their app. Currently, app providers have no formal responsibility to ensure and communicate medical evidence of their effectiveness. One aspect of ensuring this quality is building and evaluating apps in collaboration with health care professionals. Our review found that only in 11 of 42 studies were health care professionals involved in usability evaluation. Currently, it is the technology companies rather than hospitals, clinics, or doctors that are the most frequent publishers of health care apps [76], and there is a lack of involvement from health professionals in these apps [77,78].

Equally, there is a need to involve patients in the design and evaluation of mHealth apps, for example, to ensure the relevance of the apps and to obtain the experiences, beliefs, and preferences of the intended users. Most of the studies in our review of the literature involved patients; however, it remains an HCI challenge to develop ways in which to foster relevant contributions from often vulnerable patient user groups to complex design processes [79].

#### Standardize a Questionnaire for Mobile Health Apps

As in our review, Perez-Jover et al [80] found that usability evaluation practices in mHealth varied substantially. Accordingly, McFay et al [81] found a lack of best practices or standards for evaluating mHealth apps and behavioral change technologies. In a review, they found that self-developed, nonvalidated evaluation checklists were the most common evaluation method. The lack of validation casts doubt on the reliability of the results. In this review, we found that questionnaire was the most common data collection technique of the included studies; however, researchers either used standard questionnaires, such as SUS or USE, which were not specifically designed for the mental health domain, or adapted a standard questionnaire or developed a new one. Owing to the great variety of the questionnaires, there is a need to establish a common standardized usability questionnaire targeted specifically at mHealth mental health apps.

#### Foster Increased Collaboration Between Health Care and Computer Science Professionals in Mobile Health Development

Our review found that there was limited collaboration between computer science professionals and health care professionals in mHealth development. Many of the studies were carried out solely by health care professionals, and usability was evaluated in a summative manner. There is reason to believe that even closer collaboration between health care and computer science experts in the usability evaluation of mHealth apps will increase the quality of the evaluation interpretations, especially for formative evaluations.

### Limitations

There are some limitations to this study. One of these is that the study was restricted to mobile technologies, whereas several mHealth intervention technologies are available on other platforms, such as the Web for PC, and how usability is evaluated for these technologies is also important. A second limitation to this review is that we did not download and test any of the mHealth apps referred to in the reviews. Reading about an app gives a different impression than interacting with the app itself and has consequences for how we perceive the following usability evaluation, potentially limiting our understanding of this work. A third limitation pertains to the division between academically driven mHealth apps and the much larger portion developed by the technology industry as reviewing the literature for usability evaluation practices through academic databases resulted in only finding academic studies, which, in turn, influenced the usability evaluation practices we observed. Therefore, we have less knowledge of the usability evaluation practices in industry.

### Conclusions

Based on the call for evidence of their effectiveness in the plethora of mHealth intervention technologies, this study provides a detailed account of how evidence is being gathered in the form of usability evaluations from the perspective of computer science and HCI, including how users feature in the evaluation, which study objectives and outcomes are stated, which research methods and techniques are used, and what the notion of mobility features is in mHealth apps. The most common reasons for developing mobile mental health apps provided in the studies were the availability of mobile devices to users, their popularity, and device affordances. Most studies described their methods as trials gathered data from a small sample size and carried out a summative evaluation using a single questionnaire, indicating that usability evaluation was not the main focus. The extent to which a mobile mental health intervention is able to meet expectations and needs was linked to the effectiveness, efficiency, and satisfaction of such programs and thereby its usability [82]. Evidence from this literature review also indicated that almost all studies received positive feedback from their participants who found the evaluated tools useful. However, further research is required to investigate the effects of usability levels of mobile mental health apps on outcomes of an intervention. As many of the studies described using an adapted version of a standard usability questionnaire, there is a need to develop a standardized mHealth usability questionnaire, which is a goal of future research.

## Abbreviations

HCI: human-computer interaction
mHealth: mobile health
SUS: System Usability Scale
USE: Usefulness, Satisfaction, and Ease of Use
WHO: World Health Organization
